# Amyloid β Oligomers Increase ER-Mitochondria Ca^2+^ Cross Talk in Young Hippocampal Neurons and Exacerbate Aging-Induced Intracellular Ca^2+^ Remodeling

**DOI:** 10.3389/fncel.2019.00022

**Published:** 2019-02-08

**Authors:** Maria Calvo-Rodriguez, Elena Hernando-Perez, Lucia Nuñez, Carlos Villalobos

**Affiliations:** ^1^Instituto de Biología y Genética Molecular (IBGM), Consejo Superior de Investigaciones Científicas (CSIC) and Universidad de Valladolid, Valladolid, Spain; ^2^Departamento de Bioquímica y Biología Molecular y Fisiología, Universidad de Valladolid, Valladolid, Spain

**Keywords:** calcium, Alzheimer’s disease, aging, endoplasmic reticulum, mitochondria, store-operated calcium entry, hippocampal neurons

## Abstract

Alzheimer’s disease (AD) is the most common neurodegenerative disorder and strongly associated to aging. AD has been related to excess of neurotoxic oligomers of amyloid β peptide (Aβo), loss of intracellular Ca^2+^ homeostasis and mitochondrial damage. However, the intimate mechanisms underlying the pathology remain obscure. We have reported recently that long-term cultures of rat hippocampal neurons resembling aging neurons are prone to damage induced by Aβ oligomers (Aβo) while short-term cultured cells resembling young neurons are not. In addition, we have also shown that aging neurons display critical changes in intracellular Ca^2+^ homeostasis including increased Ca^2+^ store content and Ca^2+^ transfer from the endoplasmic reticulum (ER) to mitochondria. Aging also promotes the partial loss of store-operated Ca^2+^ entry (SOCE), a Ca^2+^ entry pathway involved in memory storage. Here, we have addressed whether Aβo treatment influences differentially intracellular Ca^2+^ homeostasis in young and aged neurons. We found that Aβo exacerbate the remodeling of intracellular Ca^2+^ induced by aging. Specifically, Aβo exacerbate the loss of SOCE observed in aged neurons. Aβo also exacerbate the increased resting cytosolic Ca^2+^ concentration, Ca^2+^ store content and Ca^2+^ release as well as increased expression of the mitochondrial Ca^2+^ uniporter (MCU) observed in aging neurons. In contrast, Aβo elicit none of these effects in young neurons. Surprisingly, we found that Aβo increased the Ca^2+^ transfer from ER to mitochondria in young neurons without having detrimental effects. Consistently, Aβo increased also colocalization of ER and mitochondria in both young and aged neurons. However, in aged neurons, Aβo suppressed Ca^2+^ transfer from ER to mitochondria, decreased mitochondrial potential, enhanced reactive oxygen species (ROS) generation and promoted apoptosis. These results suggest that modulation of ER—mitochondria coupling in hippocampal neurons may be a novel physiological role of Aβo. However, excess of Aβo in the face of the remodeling of intracellular Ca^2+^ homeostasis associated to aging may lead to loss of ER—mitochondrial coupling and AD.

## Introduction

Alzheimer’s disease (AD) is the most common form of dementia, accounting for 60%–80% of all dementia cases. Despite of all the efforts, the mechanisms underlying the pathology are still unknown. The two main hallmarks of AD are extracellular plaques composed of amyloid β (Aβ) peptide and intracellular neurofibrillary tangles composed of hyper-phosphorylated and misfolded tau. Most AD cases are sporadic (late-onset AD) and affect elderly people, but some cases (around 5%) are inherited in an autosomal dominant fashion, caused by mutations in the genes that encode presenilin1 (PS1), PS2 and amyloid precursor protein (APP; early-onset AD or familial AD). One of the most accepted hypothesis of AD is the “amyloid cascade hypothesis,” which postulates that the deposition of the Aβ is a central event to the pathology of AD (Hardy and Selkoe, 2002). This hypothesis considers that the pathology of AD is caused by an abnormal processing of the protein Aβ, occasioned by a reduction in its clearance or by an increase in its production. Most AD cases are sporadic, being aging the most important risk factor for the disease. During aging and, particularly, in neurodegenerative diseases, the systems that regulate intracellular Ca^2+^ homeostasis are affected, leading to synaptic dysfunction, plasticity detriment and neuronal degeneration. Consequently, a wide amount of studies has shown that the disturbance of Ca^2+^ homeostasis is associated with the neurotoxicity that occurs in AD (Mattson, 2007; Bezprozvanny, 2009; Berridge, 2010). The “Ca^2+^ hypothesis of AD” (Khachaturian, 1989) postulates that the activation of the amyloidogenic pathway induces a remodeling of the neuronal Ca^2+^ signaling, disturbing the normal Ca^2+^ homeostasis and therefore, the mechanisms responsible for learning and memory. In addition, oxidative stress, alterations in mitochondrial energetic metabolism and protein aggregation, such as Aβ in AD, affect negatively the intracellular Ca^2+^ homeostasis. Recent studies have also shown that impaired function of intracellular organelles such as the endoplasmic reticulum (ER) and mitochondria plays an important role in the regulation of Ca^2+^ during aging and AD (Toescu and Verkhratsky, 2003). In this manner, PSs, located in the membrane of the ER, have been associated to familial AD. They are integral membrane proteins that form the catalytic core of the γ-secretase complex that cleaves APP. Mutations in PSs increase the production of Aβ_1–42_ form or reduce the production of Aβ_1–40_ (Borchelt et al., 1996; Lemere et al., 1996). In addition, PSs are believed to form low conductivity channels, being involved in the formation of the *leak* channels of the ER, releasing Ca^2+^ in a passive manner from the ER to the cytoplasm (Tu et al., 2006). Mutations in PSs result in an increased release of Ca^2+^ from the ER (Nelson et al., 2007), probably due to a loss of function of the leak channel. The other key player of intracellular Ca^2+^ homeostasis, mitochondria, are dynamic organelles that generate ATP and contribute to many cellular functions. They play a main role in apoptotic signaling, lipid synthesis and buffering of intracellular Ca^2+^. Many reports have also proposed mitochondrial dysfunction in AD. In this manner, dysfunction of mitochondrial bioenergetics (Atamna and Frey, 2007; Yao et al., 2009), increased fission and decreased fusion (Wang et al., 2009; Santos et al., 2010), morphological changes (Hirai et al., 2001; DuBoff et al., 2013; Xie et al., 2013) and redistribution of mitochondria (Kopeikina et al., 2011) have been extensively reported.

ER and mitochondria are connected through the mitochondria-associated ER membranes (MAMs). They play a central role in the processes that occur between ER and mitochondria, such as communication between the two organelles, which includes Ca^2+^ transport. When the Ca^2+^ from the ER is released to the cytoplasm, part of this Ca^2+^ is taken up by mitochondria, acting as a buffer of Ca^2+^ (Szabadkai et al., 2003). If normal flux is affected, the amount of Ca^2+^ taken up by mitochondria could be increased. If there is mitochondrial Ca^2+^ overload, the mitochondrial permeability transition pore (PTPm) will open, mitochondrial membrane potential will collapse and proapoptotic factors such as cytochrome c will be released, activating the caspase pathway and triggering apoptosis. In addition, an excessive Ca^2+^ increase will enhance the production of reactive oxygen species (ROS), that will also contribute to the opening of the PTPm (Berridge, 2010).

Alteration of the correct MAM function has been previously shown in AD (Müller et al., 2018). In this manner, increased connectivity ER-mitochondria was found in human fibroblasts from patients with familiar AD mutations as well as in fibroblasts from patients with sporadic AD (Area-Gomez et al., 2012; Area-Gomez and Schon, 2017). Our group has also previously reported that Aβ oligomers (Aβo), unlike the fibrils, promote the entry of Ca^2+^ into the cell, causing mitochondrial Ca^2+^ overload and cell death by apoptosis (Sanz-Blasco et al., 2008; Calvo-Rodríguez et al., 2016a). Also, exposure of primary hippocampal neurons to conditioned media containing Aβo increased the contact points ER-mitochondria. In addition, these media also increased ER-mitochondria Ca^2+^ transfer in neuroblastoma cells (Hedskog et al., 2013). However, the effects of oligomers in the context of aging has not been addressed. Moreover, this study has never been performed in primary neurons.

We have previously described an intraorganellar Ca^2+^ remodeling with aging, involving ER-mitochondria cross-talk and loss of store-operated Ca^2+^ entry (SOCE) in rat hippocampal neurons (Calvo-Rodríguez et al., 2016b). Most studies on mechanisms underlying the pathology of AD use transgenic animal models that develop the pathology at early ages (i.e., around 6 months of age), cells from AD patients that are therefore already diagnosed, or cell lines exposed to Aβ peptides. In consequence, the alterations in the intraorganellar Ca^2+^ remodeling in AD in the context of aging have not been described, and the full spectrum of mechanisms through which Ca^2+^ homeostasis is altered in AD in aging is still missing. Based on this, we decided to study whether the soluble oligomers of the most toxic peptide involved in AD, Aβ_1–42_ (Aβo), are able to alter: (1) resting levels of cytosolic free Ca^2+^ concentration ([Ca^2+^]_cyt_); (2) SOCE; (3) the content and release of Ca^2+^ from the internal stores, as well as; (4) the Ca^2+^ transfer from ER-to mitochondria; (5) the interaction ER-mitochondria; and (6) the expression of the mitochondrial Ca^2+^ uniporter (MCU) and inositol trisphosphate receptors (IP_3_Rs) in young and aged hippocampal neurons. In order to model aging, we have employed an *in vitro* culture of hippocampal rat neurons maintained for several days *in vitro* (DIV).

Primary rat hippocampal neurons in long-term culture have been previously used as a model of aging by our group and others (Porter et al., 1997; Brewer et al., 2007; Calvo-Rodríguez et al., 2016b, 2017). Importantly, some of the changes that occur in aging are mimicked by this *in vitro* model of aging, such as accumulation of ROS, lipofuscin granules, heterochromatic foci, activation of the Jun N-terminal protein kinase (pJNK) and p53/p21 pathways and changes in NMDA receptor expression (Sodero et al., 2011; Calvo et al., 2015a). By using fluorescence Ca^2+^ imaging of fura-2 loaded cells, we have evaluated the change in the Ca^2+^ content in the ER, SOCE, the Ca^2+^ depletion from ER induced by different physiological agonists and the coupling ER-mitochondria, in young (4–8 DIV, short-term) and aged (15–21 DIV, long term) hippocampal neurons cultured *in vitro* and exposed to Aβo as a model of AD and *in vitro* aging.

## Materials and Methods

### Animals and Reagents

Wistar rat pups (newborn P0–1) were obtained from the Valladolid University animal facility. All animals were handled according to the ethical standard of Valladolid University under protocols approved by the animal housing facility and in accordance with the European Convention 123/Council of Europe and Directive 86/609/EEC. Fura-2/AM and Rhod-5N are from Invitrogen (Barcelona, Spain). Fetal bovine serum (FBS) is from Lonza (Barcelona, Spain). Horse serum, neurobasal medium, HBSS medium, B27, L-glutamine and gentamycin are from Gibco (Barcelona, Spain). Papain solution is from Worthington (Lakewood, NJ, USA). The poly-D-lysine and annexin V are from BD (Madrid, Spain). DNase I and antibody against the MCU are from Sigma (Madrid, Spain). IP_3_R1 and IP_3_R2 primary antibodies are from Santa Cruz Biotechnology (Dallas, TX, USA). IP_3_R3 primary antibody is from BD Transduction Laboratories (Madrid, Spain). ER tracker, mitotracker, tetramethylrhodamine, methyl ester (TMRM) and CM-H2DCFDA are from ThermoFisher Scientific. Aβ_1–42_ peptide is from Bachem AG (Bubenforf, Switzerland). Other reagents and chemicals were obtained either from Sigma or Merck.

### Primary Hippocampal Neuron Culture

Hippocampal neurons were prepared from Wistar rat pups under sterile conditions as reported by Brewer et al. (1993) with further modifications by Pérez-Otaño et al. (2006). Briefly, newborn rat pups were decapitated and, after brain removal, meninges were discarded and hippocampi were separated from cortex. Hippocampal tissue was cut in small pieces, transferred to papain solution (20 u./ml) and incubated at 37°C for 30 min. After 15 min, DNase I (50 μl/ml) was added. Tissue pieces were washed with Neurobasal medium and cell suspension was obtained using a fire-polished pipette in Neurobasal medium supplemented with 10% FBS. Cells were centrifuged at 160 *g* for 5 min and pellet was suspended in Neurobasal medium. Hippocampal cells were plated onto poly-D-lysine-coated, 12 mm diameter glass coverslips at 30 × 10^3^ cells/dish (plating density, 169 cells/mm^2^), and grown in Neurobasal media supplemented with L-glutamine (2 mM), gentamicin (1 μg/ml), 2% B27 and 10% FBS, maintained in a humidified 37°C incubator with 5% CO_2_ without further media exchange. Cells were cultured for 4–8 DIV for young cultures, and 15–21 DIV for aged cultures. Other details have been reported in detail elsewhere (Calvo et al., 2015a; Calvo-Rodríguez et al., 2015).

### Aβ_1–42_ Oligomers Preparation

Aβ_1–42_ oligomers (Aβo) were prepared as previously reported by a new procedure (Caballero et al., 2016). Briefly, Aβ_1–42_ peptide was initially solved at 1 mM concentration in iced cold hexafluoroisopropanol (HFIP), and separated into aliquots in sterile microcentrifuge tubes. The solution was then incubated for 2 h at room temperature (RT) to allow monomerization of the peptide. HFIP was removed under vacuum in a speed vacuum (800 *g* × 10 min at RT), and the peptide film was stored desiccated at −20°C. For aggregation, the peptide was first suspended in dry DMSO at 5 mM concentration. For complete suspension of the peptide, it was subjected to ultrasounds for 10 min, aliquoted in propylene non-siliconized tubes, and stored at −20°C until use. MEM (Gibco) medium supplemented with 0.5 mg/ml Fe^2+^, 0.5 mg/ml Cu^2+^ and 0.5 mg/ml Zn^2+^ was added to bring the peptide to a concentration of 80 μM. Finally, it was incubated at 37°C for 24 h. For experiments, Aβ_1–42_ was solved in the culturing media to 1 μM final concentration. Composition of these oligomers has been characterized previously (Caballero et al., 2016; Calvo-Rodríguez et al., 2016b).

### Effects of Aβo on Apoptosis in Young and Aged Neurons

For apoptosis, hippocampal neurons were exposed to either vehicle or Aβo 1 μM for 24 h. Cells were then washed with phosphate buffered saline (PBS) and apoptosis was evaluated using annexin V (1:20, 10 min) in annexin binding buffer 1× (in mM) NaCl, 140; CaCl2, 2.5; Hepes, 10 (pH 7.4) and assessed by fluorescence microscopy using a Nikon Eclipse TS100 microscope (objective 40×).

### Fluorescence Imaging of Cytosolic Free Ca^2+^ Concentration

Hippocampal cells were cultured for 4–8 DIV (short-term) or 15–21 DIV (long-term) and exposed to either vehicle (supplemented MEM) or 1 μM Aβo for 24 h before the experiment. The day of the experiment, hippocampal cells were washed in standard external medium (SEM) containing (in mM) NaCl 145, KCl 5, CaCl_2_ 1, MgCl_2_ 1, glucose 10 and Hepes/NaOH 10 (pH 7.4). Then cells were incubated in the same medium containing fura-2 (4 μM) for 60 min at RT in the dark. Then coverslips were placed on the perfusion chamber of a Zeiss Axiovert 100 TV, perfused continuously with the same pre-warmed medium at 37°C and epi-illuminated alternately at 340 and 380 nm using a filter wheel. Light emitted at 520 nm was recorded every 1–5 s with a Hamamatsu ER camera (Hamamatsu Photonics France). Pixel by pixel ratios of consecutive frames were captured and cytosolic Ca^2+^ concentration ([Ca^2+^]_cyt_) values from regions of interest (ROIs) corresponding to individual neurons were averaged and expressed as the ratio of fluorescence emission following excitation at 340 and 380 nm as reported in detail previously (Sanz-Blasco et al., 2008; Calvo et al., 2015b). For estimation of Ca^2+^ increase, responses (calculated as area under curve, A.U.C.) were averaged from responsive neurons (selected by their morphology different from glial cells in the brightfield). Fraction was calculated by dividing responsive cells for the total cell number in the field, considering responsive cells the ones showing a change in the slope of the Ca^2+^ recording after application of the stimulus.

For measurements of SOCE, fura-2-loaded cells were treated with the sarcoplasmic and ER Ca^2+^ ATPase (SERCA) pump blocker thapsigargin (1 μM) for 15 min in the same SEM devoid of extracellular Ca^2+^ before imaging as reported elsewhere (Calvo-Rodríguez et al., 2016b). Then cells were subjected to fluorescence imaging and stimulated with 5 mM Ca^2+^ to monitor the SOCE-dependent rise in [Ca^2+^]_cyt_. Recordings were made in the presence of TTX to prevent activation of voltage-gated Ca^2+^ channels by connected neurons.

For estimation of Ca^2+^ store content, the rise in [Ca^2+^]_cyt_ induced by low concentrations of the Ca^2+^ ionophore ionomycin (400 nM) added in the absence of extracellular Ca^2+^ was monitored as reported previously (Villalobos and García-Sancho, 1995).

### Fluorescence Imaging of Mitochondrial Free Ca^2+^ Concentration

Hippocampal cells cultured for 4–8 DIV (short-term) or 15–21 DIV (long-term) were exposed to either vehicle or 1 μM Aβo for 24 h before the experiment. The day of the experiment, hippocampal cells were washed in SEM and incubated in the same medium containing Rhod-5N AM (1 μM) for 30 min at RT in the dark and washed in SEM for additional 30 min. Then coverslips were placed on the perfusion chamber of a Zeiss Axiovert 100 TV, perfused continuously with the same pre-warmed medium at 37°C and epi-illuminated in the red channel (551 nm). Light emitted at 576 nm was recorded every 5 s with a Hamamatsu ER camera (Hamamatsu Photonics France). Pixel by pixel ratios of consecutive frames were captured and mitochondrial Ca^2+^ concentration ([Ca^2+^]_mit_) values from ROIs corresponding to individual neurons were averaged and expressed as normalized fluorescence emission (F/F_0_). For estimation of Ca^2+^ increase, responses (calculated as amplitude of the peak) were averaged from responsive neurons (selected by their morphology different from glial cells in the brightfield). Fraction was calculated by dividing responsive cells for the total cell number in the field, considering responsive cells the ones showing a change in the slope of the Ca^2+^ recording after application of the stimulus.

### Immunofluorescence of the Mitochondrial Calcium Uniporter (MCU) and IP_3_ Receptors

Hippocampal cells at different culture periods and exposed to either vehicle or 1 μM Aβo for 24 h were washed with phosphate buffered saline (PBS), fixed with p-formaldehyde 4% and incubated with antibodies against MCU (1:200) and IP_3_ receptors IP_3_R1 (1:50), IP_3_R2 (1:50) and IP_3_R3 (1:50) at 4°C overnight. Immunopositive cells were revealed using Alexafluor 488-tagged antibodies (1:300). Optical density was measured in selected ROIs corresponding to individual neurons using ImageJ software. Further details have been reported elsewhere (Calvo-Rodríguez et al., 2016b).

### ER-Mitochondria Colocalization

Cells in every condition were stained for 10 min with 200 nM MitoTracker green and ERTracker red and imaged directly using a confocal microscope. High resolution images of cells were recorded by using a Leica TCS SP5 confocal microscope (Leica Microsystems, Mannheim, Germany). The Mitotracker and ER-tracker were excited with 488 and 543 laser lines, respectively, and emission was acquired with a charged CCD camera. The images were analyzed in LAS AF Lite software (Leica Microsystems, Mannheim, Germany). Background was subtracted from all images. Colocalization analysis for the two markers was performed on Z-stacks acquired with steps of 0.8 μm. For colocalization analysis, green and red channel images were acquired independently. Analysis were carried out on single-plane images using ImageJ plug-ins. Manders2 and Pearson’s coefficients were calculated applying the ImageJ Colocalization analysis plug-in.

### Mitochondrial Membrane Potential

For mitochondrial membrane potential measurements, hippocampal neurons were exposed to either vehicle or Aβo 1 μM for 24 h. Then, cells were washed in SEM and loaded with the mitochondrial potential probe (TMRM, 10 nM) for 30 min at RT in the dark. Then coverslips containing cells were placed on a Zeiss Axiovert 100 TV inverted microscope and subjected to fluorescence imaging. Fluorescence images were captured with the rhodamine filter set with a Hamamatsu ER-Orca fluorescence camera as reported previously (Calvo-Rodríguez et al., 2016b).

### Generation of Reactive Oxygen Species (ROS)

For ROS generation measurements, hippocampal neurons were exposed to either vehicle or Aβo 1 μM for 24 h. Then, cells were washed in SEM and loaded with the ROS probe 5-(and-6)-chloromethyl-2′,7′-dichlorodihydrofluorescein diacetate (CM-H2DCFDA 2 μM) for 40 min at RT in the dark. Then coverslips containing cells were placed on a Zeiss Axiovert 100 TV inverted microscope and subjected to fluorescence imaging. Fluorescence images were captured with the FITC filter set with a Hamamatsu ER-Orca fluorescence camera.

### Statistics

Changes in fluorescence ratio are expressed as area under the curve (AUC) and maximum increase in ratio (Δratio). Calculation of AUC and Δratios were performed using Origin Lab 7.0. Data are presented as mean ± SEM. When only two means were compared, Student’s *t*-test was used. For more than two groups, statistical significance of the data was assessed by one-way or multifactorial analysis of variance (ANOVA), depending on the number of factors considered. Differences were considered significant at *p* < 0.05, where 0.05 is the signification level.

## Results

### Aβ Oligomers Induce Apoptosis Only in Aged Neurons

**Figure 1:**
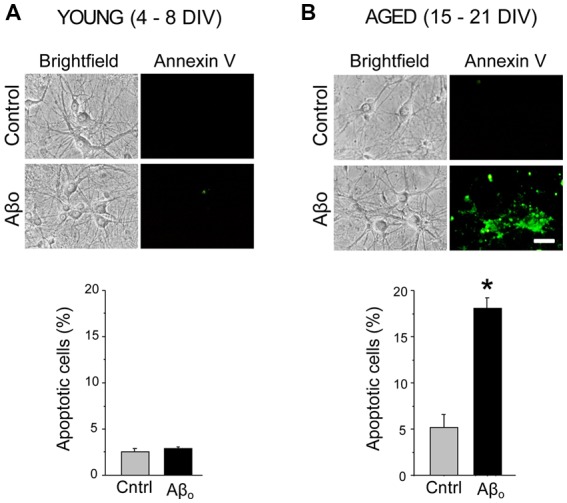
Amyloid β peptide oligomers (Aβo) induce apoptosis only in aged neurons. Short-term (young) and long-term (aged) hippocampal cultures were exposed for 24 h Aβo (1 μM) or vehicle. Then, the relative abundance (percent, %) of apoptotic neurons was assessed by incubation with annexin V. Pictures show representative bright field cultures of short-term **(A)** and long-term cultures **(B)** of rat hippocampal neurons. Bars represent mean ± standard external medium (SEM) percentage of apoptotic cells per condition. *n* = 339 and 330 cells from three independent experiments for young neurons **(A)** and 452 and 417 cells from four independent experiments, respectively for aged neurons **(B)**. *p < 0.05 compared to control group.

We tested the effects of Aβ_1–42_ oligomers (Aβo) on the rate of apoptosis in rat hippocampal neurons cultured for 4–8 DIV representing young neurons, or in neurons cultured for 15–21 DIV that represent many characteristics of aged neurons as reported previously (Calvo-Rodríguez et al., 2016a). Short-term (4–8 DIV) and long-term (15–21 DIV) cultured hippocampal neurons were exposed for 24 h to 1 μM Aβo or to vehicle (supplemented MEM, see “Materials and Methods” section). This latter condition was taken as control group. Figure 1 shows that, in resting conditions, only less than 3% of the young neurons display apoptosis. Aβo do not increase the rate of apoptosis in these cultured cells. About 5% of aged neurons display apoptosis in basal conditions. In contrast, Aβo increase the percent of apoptotic cells by nearly four-fold. These results confirm our previous results reported elsewhere (Caballero et al., 2016; Calvo-Rodríguez et al., 2016a) and indicate that Aβo toxicity depends on the time in culture of rat hippocampal neurons.

In our hands, cultured rat hippocampal cells display a mix of neurons and glial cells and their relative abundance changes with aging in culture mostly because glial cells proliferate while neuron cells do not. The fraction of neurons amounts 60% at 5 DIV, decreases to 23% at 14 DIV and decreases further to only 15% at 20 DIV. Therefore, for monitoring of effects of Aβo in neurons, it is convenient to carry out measurements at the single cell level in identified neurons. Fortunately, hippocampal neurons can be easily identified and distinguished from astrocytes or glial cells according to morphometric characteristics for the expert eye. Next, we addressed whether treatment of rat hippocampal neurons with Aβo promote differential changes in intracellular Ca^2+^ homeostasis in young and aged neurons.

### Aβ Oligomers Increase Resting [Ca^2+^]_cyt_ and Synchronous Ca^2+^ Oscillations in Aged Neurons

**Figure 2:**
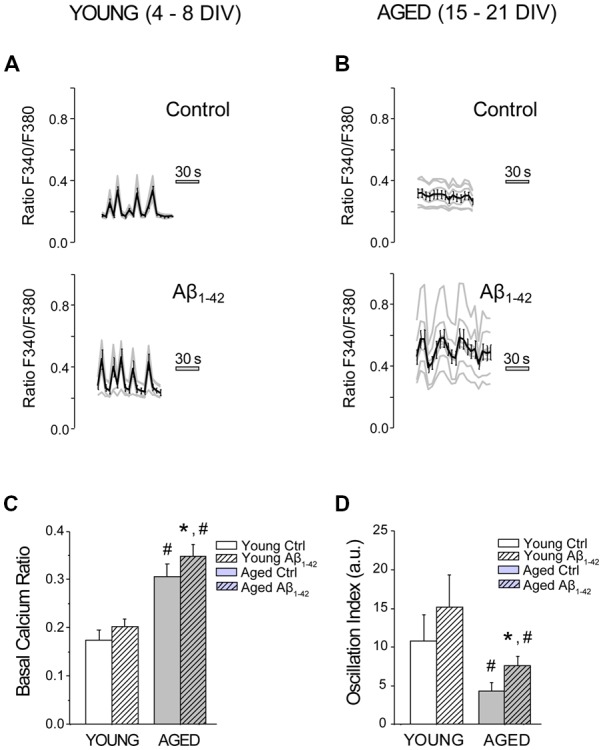
The exposure of aged hippocampal neurons to Aβo increases basal Ca^2+^ and synchronous and spontaneous Ca^2+^ oscillations. Hippocampal neurons cultured for different days *in vitro* (DIV), were exposed to vehicle or Aβo (1 μM, 24 h), and [Ca^2+^]_cyt_ was monitored by fluorescence imaging using fura-2. **(A,B)** Traces show individual cells (gray lines) and mean ± SEM (black line) of the increase in [Ca^2+^]_cyt_ prior to any stimulus in young **(A)** and aged **(B)** cultured hippocampal neurons exposed to either Aβo (1 μM) or vehicle. **(C,D)** Bars represent mean ± SEM of basal Ca^2+^ (ratio F340/F380; **C**) and the oscillation index **(D)** for young and aged neurons, treated in absence (ctrl) of presence of the Aβo (Aβ_1–42_, hatched bars). *n* = 113, 97, 131 and 169 neurons from four to six independent experiments, respectively. *p < 0.05 compared to control group. ^#^*p* < 0.05 compared to the young ones.

We have tested the effects of chronic treatment of Aβo on resting [Ca^2+^]_cyt_, a parameter previously reported to vary as hippocampal neurons age in culture (Calvo et al., 2015a). Short-term (4–8 DIV) and long-term (15–21 DIV) cultured hippocampal neurons were exposed for 24 h to 1 μM Aβo or to vehicle (supplemented MEM). The effects of treatments were assessed by calcium imaging of fura-2 loaded neurons. Neurons were clearly distinguished from surrounding glial cells by their morphometric characteristics as previously reported (Calvo-Rodríguez et al., 2016b). Figure 2 shows that short-term cultured, rat hippocampal neurons display spontaneous, synchronous [Ca^2+^]_cyt_ oscillations. Notice that the average recording runs in parallel to the recordings of individual cells, indicating that oscillations are synchronous. These synchronous oscillations reflect synchronous synaptic activity and the formation of neural networks *in vitro* (Nunez et al., 1996; Calvo-Rodríguez et al., 2016b). Long-term cultured rat hippocampal neurons exposed to Aβo (1 μM, 24 h) display increased [Ca^2+^]_cyt_ levels (resting level) when compared to the control neurons (Figure 2C). Moreover, treatment of long-term cultured hippocampal neurons with 1 μM Aβo for 24 h increases significantly the synchronous and spontaneous [Ca^2+^]_cyt_ oscillations compared to the control group measured as oscillations index (OI), a parameter representing both the frequency and amplitude of [Ca^2+^] oscillations (Figure 2D; Nunez et al., 1996). In contrast, exposure of short-term cultured neurons to 1 μM Aβo had a little or no effect on both parameters, resting [Ca^2+^]_cyt_ and OI. These results suggest that prolonged exposure of hippocampal neurons *in vitro* to Aβo increases the excitability of the neuronal circuit, at the level of basal Ca^2+^ and spontaneous oscillations. This effect is statistically significant only in aged neurons and Aβo has no effect in this regard in young neurons.

### Aβ Oligomers Dampen Further the Decreased Store Operated Ca^2+^ Entry (SOCE) in Aged Neurons

**Figure 3:**
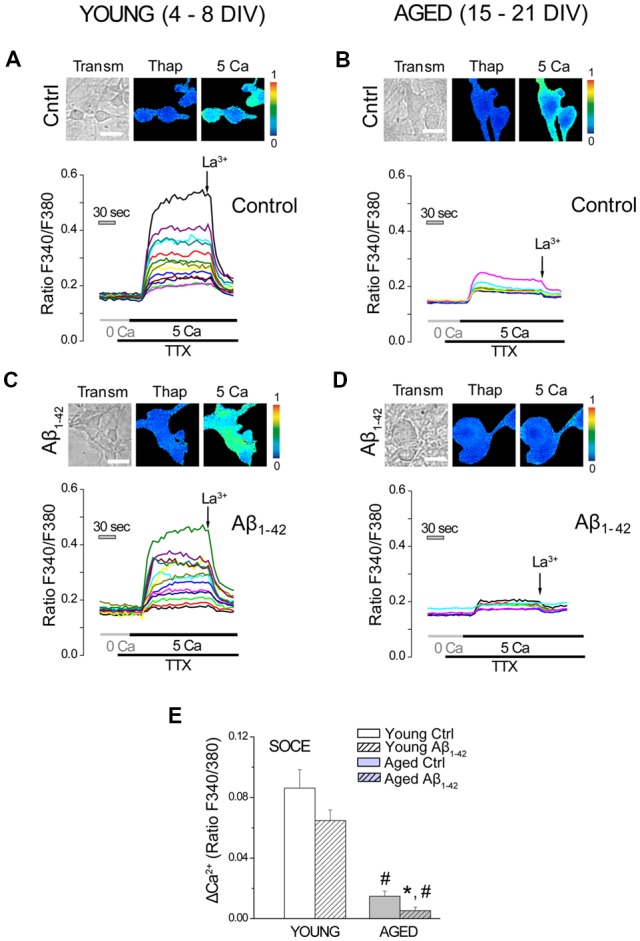
Store operated Ca^2+^ entry (SOCE) decreases in long-term cultured hippocampal neurons exposed to Aβo. Short- and long-term cultured hippocampal neurons were either exposed to vehicle (ctrl) or Aβo (1 μM, 24 h), and SOCE was monitored by fluorescence imaging of Ca^2+^. Neurons were incubated with fura-2 and treated with Tg (1 μM) for 15 min in medium devoid of Ca^2+^ (0 Ca). Subsequently, Ca^2+^ 5 mM in presence of TTX (500 nM) was added to elicit an increase in the [Ca^2+^]_cyt_. La^3+^ (100 μM) was added to block SOCE. **(A–D)** Pictures show typical fluorescence images of transmission (transm) and [Ca^2+^]_cyt_ coded in pseudocolor of thapsigargin (Thap) and after addition of 5 Ca. Pseudocolor scale is shown at right. Bars represent 10 μm. Traces are representative recordings of fluorescence ratios of individual short-term (young, 4–8, **A**,**C**) and long-term (aged, 15–21, **B,D**) cultured neurons. **(E)** Bars correspond to the averaged SOCE (mean ± SEM). *n* = 65, 84, 35 and 45 individual cells studied in three and three independent experiments, respectively. *p < 0.05 compared to control group. ^#^*p* < 0.05 compared to the young ones.

We have previously shown that SOCE, the Ca^2+^ entry pathway activated after depletion of intracellular Ca^2+^ stores, decreases with *in vitro* aging (Calvo-Rodríguez et al., 2016b). This process has been associated to a downregulation of stromal interaction molecule 1 (Stim1) and Orai1, molecular players involved in SOCE in most cell types. Here, we assessed the effect of Aβo on SOCE in our model of *in vitro* aging. Short-term and long-term cultured hippocampal neurons were exposed to either vehicle (Control) or Aβo (1 μM, 24 h), and SOCE was monitored by fluorescence imaging of [Ca^2+^]_cyt_. For this end, cultured neurons were loaded with fura-2 and treated with Tg (1 μM) for 15 min in medium devoid of extracellular Ca^2+^ (0 Ca) in order to fully deplete the intracellular Ca^2+^ stores. Subsequently, medium containing Ca^2+^ 5 mM and TTX (500 nM) was added for 5 min to activate SOCE. TTX was added to block the electrical connectivity of cultured neurons. La^3+^ (100 μM) was added later to block this pathway. Pictures in Figure 3 show typical bright field images (transm) and fluorescence images coded in pseudocolor representing [Ca^2+^]_cyt_ before (basal) and after addition of 5 mM Ca^2+^ (5 Ca). Representative recordings of fluorescence ratios of individual short-term (young, 4–8 DIV, Figures 3A,C) and long-term (aged, 15–21 DIV, Figures 3B,D) cultured neurons are also shown. Figure 3E shows bars corresponding to the averaged SOCE calculated as ΔRatioF340/F380 (mean ± SEM). Data confirm our previous results showing that SOCE is reduced significantly in *in vitro* aged neurons relative to young neurons. In addition, they show that AβO decrease SOCE further but this effect is only statistically significant in aged neurons.

### Aβ Oligomers Increases Further the Enhanced Ca^2+^ Store Content in Aged Neurons

**Figure 4:**
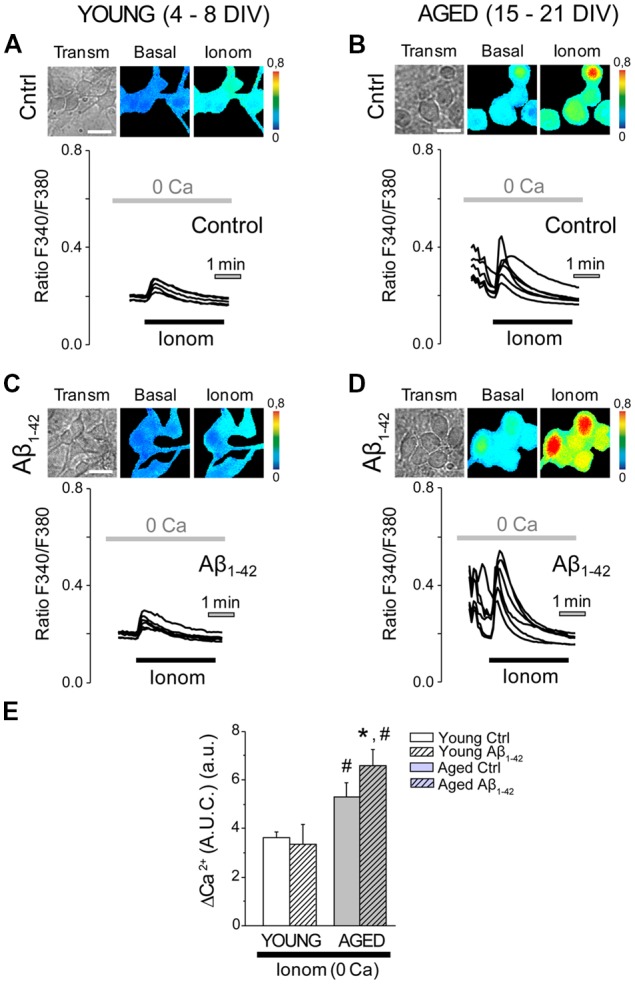
The exposure of aged hippocampal neurons to Aβo increases Ca^2+^ store content. Hippocampal neurons exposed to either Aβo (1 μM) or vehicle and loaded with fura-2 were stimulated with ionomycin (400 nm) in external medium devoid of Ca^2+^ (0 Ca) and subjected to fluorescence imaging to estimate the content of Ca^2+^ deposits. **(A–D)** Pictures show transmission (transm) and pseudocolor images of fluorescence ratios in basal conditions (basal) and after ionomycin (Ionom) stimulation in short-term (**A,C**, Young 4–8 DIV) and long-term (**B,D**, Aged 15–21 DIV) cultured hippocampal neurons not exposed (**A,B**, control) or exposed (**C,D**, Aβ_1–42_) to Aβo (1 μM, 24 h). Pseudocolor bars are shown at right. Bar represent 10 μm. Traces are representative recordings of the Ca^2+^ release induced by ionomycin (400 nM) in medium devoid of Ca^2+^ (0 Ca) in individual neurons subjected to fluorescence imaging. **(E)** Bars represent the average (mean ± SEM) in the increase of [Ca^2+^]_cyt_ in response to ionomycin (measured as area under the curve, AUC) in short-term (young) and long-term (aged) hippocampal neurons exposed to either Aβo (1 μM, 24 h) or vehicle. *n* = 55, 42, 81 and 114 individual cells from three to six independent cultures, respectively. *p < 0.05 compared to control group. ^#^*p* < 0.05 compared to the young ones.

We have reported previously that *in vitro* aging increases Ca^2+^ store content in rat hippocampal neurons (Calvo-Rodríguez et al., 2016b). Ca^2+^ store content was assessed after chronic exposure to Aβo in young and aged neurons. For this end, Ca^2+^ store content was tested using the Ca^2+^ ionophore ionomycin added in medium devoid of external Ca^2+^. This procedure has been reported previously to estimate Ca^2+^ store content in living cells (Villalobos and García-Sancho, 1995; Calvo-Rodríguez et al., 2016b). Ca^2+^ store content was monitored by fluorescence imaging in short-term and long-term cultured hippocampal neurons, exposed to Aβo (1 μM, 24 h), and compared to the controls (exposed to the vehicle for dilution of Aβo, MEM). These experiments were always carried out in medium devoid of extracellular Ca^2+^ (0 Ca), in order to avoid the entry of Ca^2+^ from the external medium. The stimulation of hippocampal neurons with 400 nM ionomycin in Ca^2+^ free medium (0 Ca; Figure 4) increased [Ca^2+^]_cyt_ in all neurons studied (measured as AUC). Exposure of the short-term cultured cells to Aβo did not modify the increase in [Ca^2+^]_cyt_ induced by ionomycin (Figures 4A,C). However, in the long-term cultured cells, chronic exposure to Aβo for 24 h increased significantly the content of the stores, as shown by the enhanced increase in the [Ca^2+^]_cyt_ induced by ionomycin in 0 Ca medium (Figures 4B,D). These results suggest that the chronic treatment of cultured hippocampal neurons with Aβo increases further Ca^2+^ store content exclusively in the long-term cultured neurons, an effect that could be related to the increase in the basal Ca^2+^ levels that were appreciated in the aged neurons exposed to the Aβo, but that does not occur in the young cultured ones.

### Aβ Oligomers Increase Further the Enhanced Release of Ca^2+^ Induced by Acetylcholine in Aged Neurons

**Figure 5:**
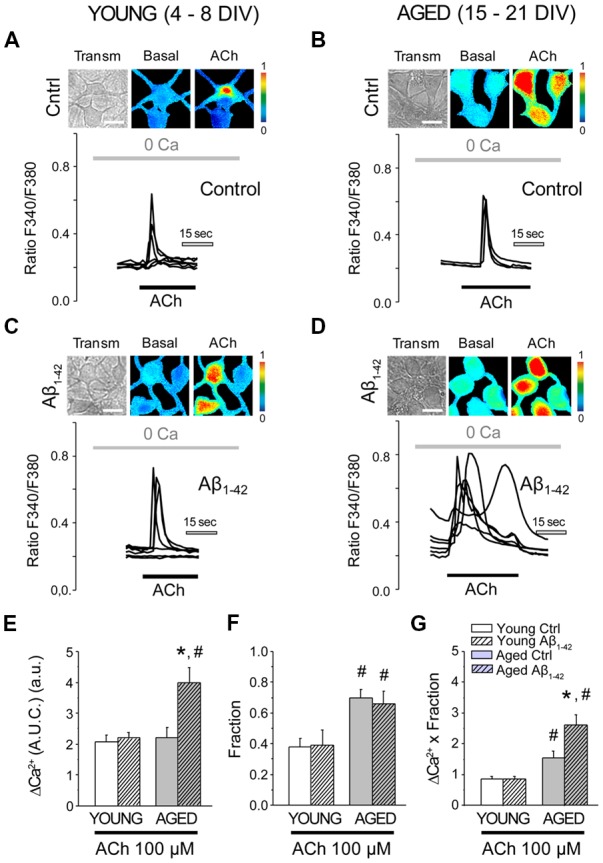
The exposure of aged hippocampal neurons to Aβo increases Ca^2+^ responses to acetylcholine (ACh) in rat hippocampal neurons. Hippocampal neurons incubated with vehicle or Aβo (1 μM, 24 h) and loaded with fura-2 were subjected to fluorescence imaging for monitoring the Ca^2+^ released by ACh in short-term and long-term cultured neurons. Cells were stimulated with ACh in Ca^2+^ free medium. **(A–D)** Pictures show transmission images (transm) and pseudocolor images of fluorescence ratios taken before (Basal) and after ACh stimulation. Pseudocolor scale is shown at right. Bars correspond to 10 μm. Traces represent significant fluorescence recordings in single neurons of the Ca^2+^ release induced by ACh (100 μM) in young and aged cultures not exposed (**A,B**, control) or exposed (**C,D**, Aβ_1–42_) to Aβo (1 μM, 24 h). **(E–G)** Bars represent the summary results for 102, 94, 39 and 59 cells from four independent cultures, respectively (mean ± SEM, AUC) of responsive cells **(E)**, the fraction of responsive cells **(F)**, and the product of both parameters **(G)**. *p < 0.05 compared to control group. ^#^*p* < 0.05 compared to the young ones.

In order to understand the functional consequences of the increase in the levels of Ca^2+^ in the intracellular stores, we then analyzed the release of Ca^2+^ from the ER that is mobilized by different agonists. The release of Ca^2+^ mediated by the IP_3_ receptor was first evaluated using the neurotransmitter acetylcholine (ACh). In order to cancel the contribution of the entry of Ca^2+^ and just focus on the release from the stores, all the experiments were carried out in the absence of free Ca^2+^ in the extracellular medium (0 Ca). Application of ACh to cultured hippocampal neurons in 0 Ca produced an increase in the [Ca^2+^]_cyt_, as described previously (Calvo-Rodríguez et al., 2016b). Figure 5 shows representative recordings in short-term and long-term cultured hippocampal neurons exposed to either Aβo (1 μM, 24 h) or vehicle, and subjected to fluorescence Ca^2+^ imaging. It can be observed that the chronic treatment with Aβo does not affect the increase in the [Ca^2+^]_cyt_ induced by ACh in short-term cultured hippocampal neurons (Figures 5A,C). In contrast, chronic exposure of long-term cultured hippocampal neurons to Aβo promoted a larger [Ca^2+^]_cyt_ response to ACh when compared to control cells (Figures 5B,D). Figures 5E–G show the quantification of the response. Bars represent the mean value of the [Ca^2+^]_cyt_ increase, represented as AUC (AUC ± SEM) of the Ca^2+^ response (Figure 5E). The fraction of cells that presented an increase in the [Ca^2+^]_cyt_ is also showed (Figure 5F). It can be observed that the fraction of cells that responded to the stimulation with ACh is higher in long-term cultured hippocampal neurons. In order to compare the response between young and aged neurons, a measure of global release of Ca^2+^ involving both parameters (Ca^2+^ released and fraction of responding cells) was calculated. For this end we multiplied the AUC of the cells that showed an increase in the [Ca^2+^]_cyt_ by the fraction of cells that responded to the stimulus. Figure 5G shows the global difference in Ca^2+^ release from the stores among the four populations. Notice that whereas aging increases the fraction of neurons responsive to ACh, treatment with Aβo enhances instead the release of Ca^2+^ in each individual cell. Thus, aging increases the fraction of neurons responsive to ACh and Aβo enhances the rise in Ca^2+^ elicited by ACh in aged neurons. No effect was observed in young neurons.

### Aβ Oligomers Increase Further the Enhanced Release of Ca^2+^ Induced by Caffeine in Aged Neurons

**Figure 6:**
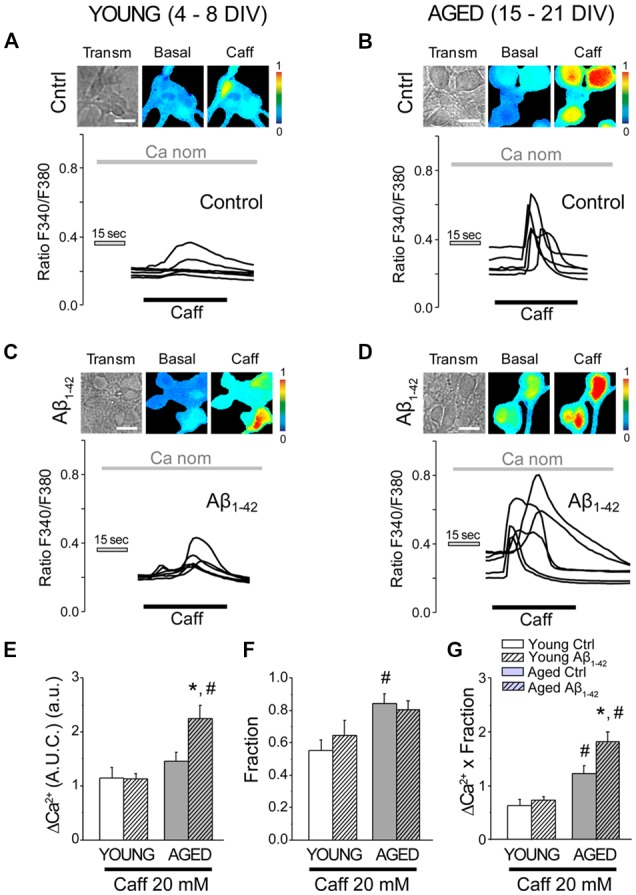
The exposure of aged hippocampal neurons to Aβo increases Ca^2+^ responses to caffeine in rat hippocampal neurons. Short-term (young, 4–8 DIV) and long-term (aged, 15–21 DIV) cultured hippocampal neurons were either exposed to vehicle or Aβo (1 μM, 24 h) and loaded with fura-2 and subjected to fluorescence imaging. Pictures show representative images of transmission (transm) and pseudocolor coded of basal and the maximum response of Ca^2+^ to caffeine in young **(A,C)** and aged cells **(B,D)**. Pseudocolor scale is shown at right. Bars correspond to 10 μm. Traces represent single-cell recordings of young and aged cells non-exposed (ctrl) or exposed (Aβ_1–42_) to Aβo (1 μM, 24 h) of the Ca^2+^ response induced by caffeine 20 mM in medium nominal Ca^2+^. **(E–G)** Bars represent mean ± SEM of the response of [Ca^2+^]_cyt_ to caffeine (expressed as AUC) of 56, 78, 94 and 105 cells from three to five independent cultures, respectively **(E)**. Panel **(F)** shows the fraction of cells that responded. Panel **(G)** represents the overall result of the multiplication of both factors. *p < 0.05 compared to control group. ^#^*p* < 0.05 compared to the young ones.

Next, we assessed the effects of Aβo on Ca^2+^ release induced by activation of ryanodine receptors (RyRs) in short-term and long-term cultured neurons. Caffeine (Caff; 20 mM) was employed as an agonist of the RyRs. Caffeine is an agonist of the RyRs, which are sensitive to Ca^2+^-induced Ca^2+^ released (CICR) processes. In this case, nominally free Ca^2+^ (without adding EGTA) was used as external cellular medium to be able to activate CICR, while minimizing the effect of Ca^2+^ entry from the extracellular medium into the cytosol. To avoid high osmotic variation following the application of 20 mM Caff, 20 mM glucose was added to the extracellular medium prior to its addition, subsequently replaced by 20 mM Caff (Calvo-Rodríguez et al., 2016b). When hippocampal neurons were stimulated by caffeine in nominally free Ca^2+^, a rapid and transient increase in the [Ca^2+^]_cyt_ could be observed (Figure 6). The bars shown in Figure 6E represent the mean ± SEM value of the increase in [Ca^2+^]_cyt_ (quantified as AUC). ER Ca^2+^ mobilized by caffeine was similar in the young neurons (exposed or not exposed to 1 μM Aβo for 24 h) and in the non-exposed cultured-aged neurons. However, in cultured-aged neurons previously exposed to Aβo (1 μM, 24 h), the increase in [Ca^2+^]_cyt_ was significantly higher than in the control group. Figure 6G shows the global increase (AUC Ca^2+^ increase × fraction) produced by stimulation with caffeine under these conditions. Therefore, Ca^2+^ release induced by both IP_3_ receptor and RyR agonists is further enhanced by Aβo treatment in aged neurons but not in young neurons.

### Aβ Oligomers Enhance ER-Mitochondria Cross Talking in Young Neurons but They Promote Opposite Effects in Aged Neurons

**Figure 7:**
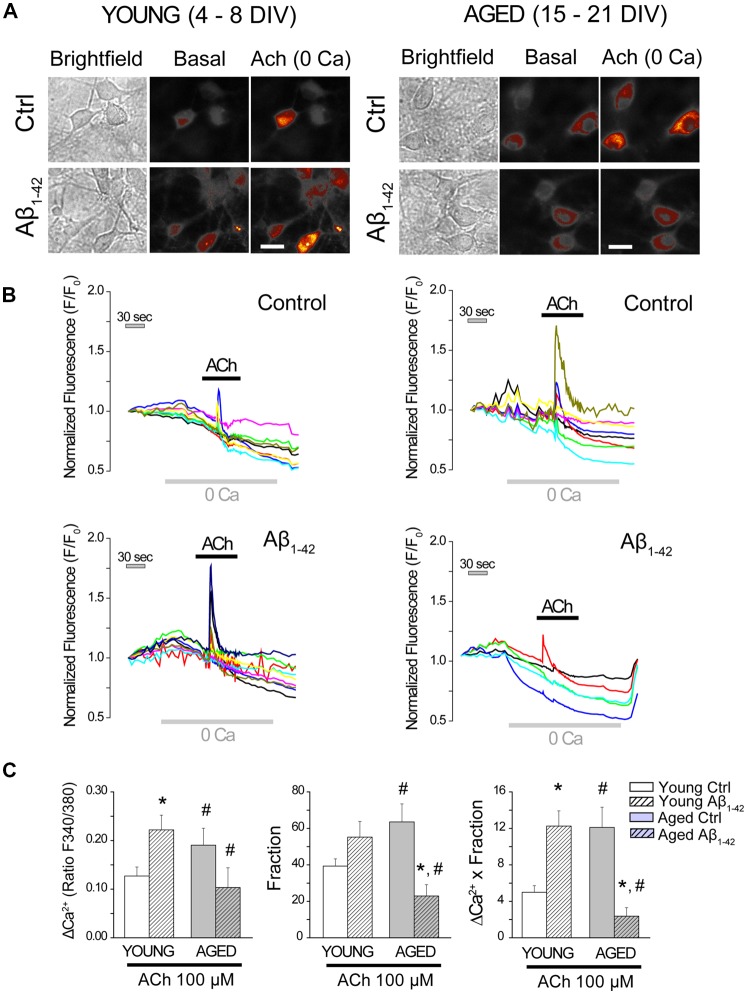
The exposure of hippocampal neurons to Aβo affects the cross-talk endoplasmic reticulum (ER)–mitochondria. Hippocampal neurons cultured for 4–8 (young) and 15–21 (aged) DIV were incubated with Rhod-5N (1 μM) and subjected to fluorescence imaging to measure [Ca^2+^]_mit_ in single cell. Cells exposed to either vehicle (control) or Aβo (1 μM, 24 h) were stimulated with the agonist ACh in medium devoid of Ca^2+^ (0 Ca). **(A)** Pictures show typical fluorescence images of transmission (transm) and Rhod-5N fluorescence in basal conditions of after stimulation with ACh (100 μM) in medium 0 Ca of short and long-term cultured hippocampal neurons. The bars represent 10 μm. **(B)** Traces represent characteristic response of [Ca^2+^]_mit_ in single cells by stimulating with ACh (100 μM) in medium 0 Ca. **(C)** Bars represent mean ± SEM of the increase in [Ca^2+^]_mit_ induced by ACh in 0 Ca, the fraction of response and the global response by taking into account both factors. The global response to ACh is significantly higher in the young neurons exposed to Aβo (1 μM, 24 h) and significantly smaller in the exposed aged ones. *n* = 44, 66, 29 and 31 individual cells studied in three and three independent experiments, respectively. *p < 0.05 compared to control group. ^#^*p* < 0.05 compared to the young ones.

Next, we tested the effects of Aβo on ER-mitochondria cross-talk, i.e., Ca^2+^ transfer from ER to mitochondria. For this end, we measured the increase in mitochondrial [Ca^2+^] ([Ca^2+^]_mit_) induced by Ca^2+^ release elicited by ACh in medium devoid of Ca^2+^ (0 Ca), in the same manner as for Figure 5. Short-term and long-term cultured hippocampal neurons were incubated either with vehicle (Control) or Aβo (1 μM, 24 h). Then cells were washed and preincubated with the mitochondrial Ca^2+^ probe Rhod-5N, a fluorescent calcium probe that targets mitochondria (de la Fuente et al., 2012), and subjected to fluorescence imaging in single neurons. Neurons treated with either vehicle (control) or Aβo were perfused with the agonist ACh in medium devoid of Ca^2+^ (0 Ca) to induce the release of Ca^2+^ from the ER. Figure 7 shows bright field and Rhod-5N fluorescence images in basal conditions and after stimulation with ACh (100 μM) in short-term (4–8 DIV) and long-term (15–21 DIV) cultured hippocampal neurons. Representative recordings of [Ca^2+^]_mit_ obtained in single cells before and after stimulation with ACh are also shown. Bars represent the quantification of the rises in [Ca^2+^]_mit_ induced by ACh in 0 Ca (left), the fraction of responsive cells (middle) and the global response by taking into account both factors (right). As shown previously using mitochondria-targeted aequorin (Calvo-Rodríguez et al., 2016b), the rise in [Ca^2+^]_mit_ induced by ACh is larger in aged neurons than in young neurons. Therefore, these results confirm our previous results and validate monitoring of [Ca^2+^]_mit_ with Rhod-5N. The larger rise in [Ca^2+^]_mit_ in aged neurons is due to both the increase in mitochondrial Ca^2+^ uptake in every cell (left panel) and the fraction of responsive cells (middle panel) corresponding to a large global ER-mitochondria cross talk in aged neurons relative to young neurons (right panel; Figure 7C). Interestingly, Aβo increased the rise in [Ca^2+^]_mit_ induced by ACh in young neurons (left panel) and this effect was not due to changes in the fraction of responsive cells (middle panel). Therefore, Aβo increase ER-cross talk in young neurons (right panel, Figure 7C). It is noteworthy that this effect cannot be due to increased ER Ca^2+^ content (Figure 4E) and is the only effect induced by Aβo that we have observed in young neurons.

It is also remarkable that the effects of Aβo on the rise in [Ca^2+^]_mit_ induced by ACh in young and aged neurons are opposite. Aβo increases Ca^2+^ transfer from ER to mitochondria in young neurons and decreases it in aged neurons. In fact, Aβo nearly abolishes the rise in [Ca^2+^]_mit_ induced by ACh in aged neurons and this effect is due to decreases in both the amount of Ca^2+^ transferred per neuron and the fraction of neurons responsive to ACh. Taken together, these results show that Aβo significantly increases ER-mitochondria cross-talk in young neurons, but they rather abolish it in aged neurons.

### Aβ Oligomers Enhance Further the Increased Expression of the Mitochondrial Ca^2+^ Uniporter in Aged Neurons

**Figure 8:**
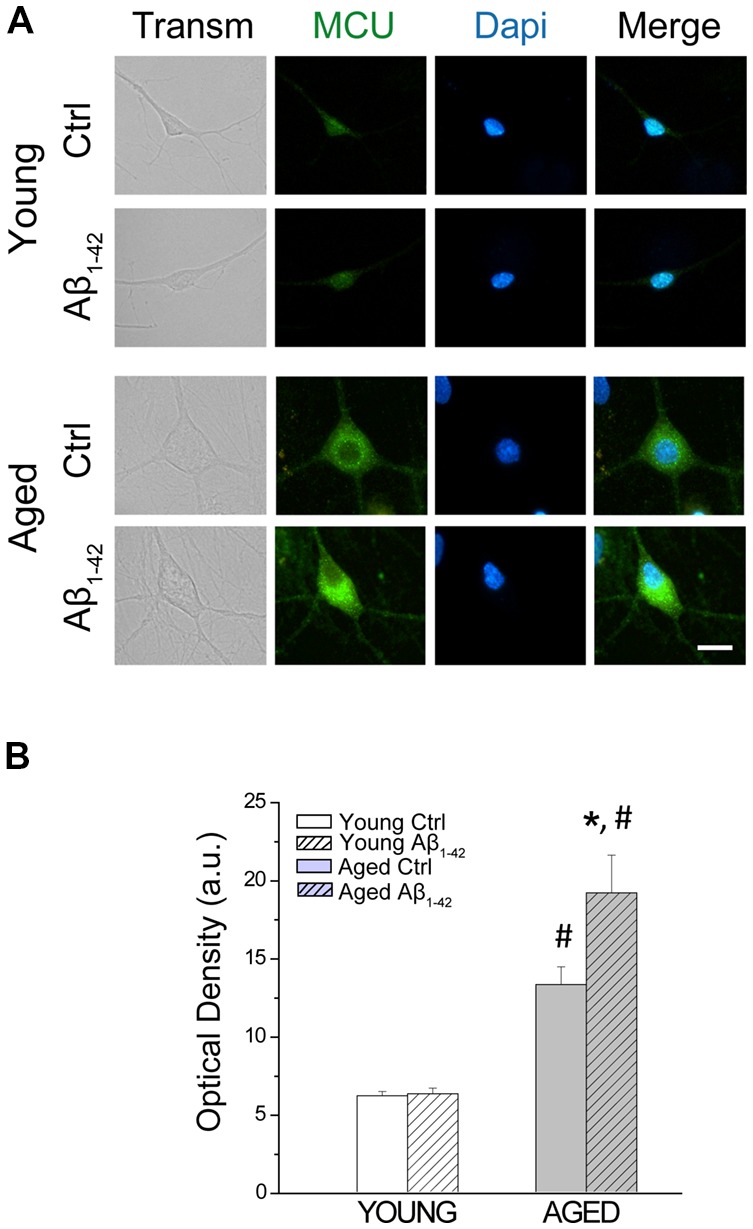
The exposure of aged hippocampal neurons to Aβo increases the expression of the mitochondrial Ca^2+^ uniporter (MCU). Immunostaining against MCU was assessed in hippocampal neurons incubated either with vehicle (ctrl) or Aβo (1 μM, 24 h). **(A)** Representative images of the fluorescence detection of MCU in short-term (young) and long-term (aged) cultured hippocampal neurons. Bar represents 10 μm and applies to all images. **(B)** Quantitative analysis of fluorescence intensity (optical density, arbitrary units) of the MCU. Bars represent average ± SEM. *n* = 112, 81, 90 and 59 cells respectively from three independent cultures. *p < 0.05 compared to control group. ^#^*p* < 0.05 compared to the young ones.

Ca^2+^ enters the mitochondria through the MCU, located into the inner mitochondrial membrane (Baughman et al., 2011; De Stefani et al., 2011). To address the opposite effects of Aβo on mitochondrial Ca^2+^ uptake in young and aged neurons, we also tested the effects of Aβo on MCU expression. Short-term and long-term cultured neurons were incubated either with vehicle (ctrl) or Aβo (1 μM, 24 h). Then, immunostaining against MCU was evaluated by immunofluorescence. Figure 8 shows representative images of the fluorescence detection of MCU in short-term (young) and long-term (aged) cultured hippocampal neurons and the quantitative analysis of fluorescence intensity (optical density, arbitrary units) of the MCU. As reported previously (Calvo-Rodríguez et al., 2016b), we found that optical density of MCU immunofluorescence is enhanced in aged neurons relative to young neurons, thus confirming our previous results. In addition, we found that Aβo significantly increased the expression of MCU in the long-term cultured hippocampal neurons but not in young neurons. These results indicate that the opposite effects of Aβo on ER-mitochondria cross talking in young and aged neurons are not due to changes in expression of MCU.

### Aging Increases Expression of IP_3_ Receptors in Rat Hippocampal Neurons

**Figure 9:**
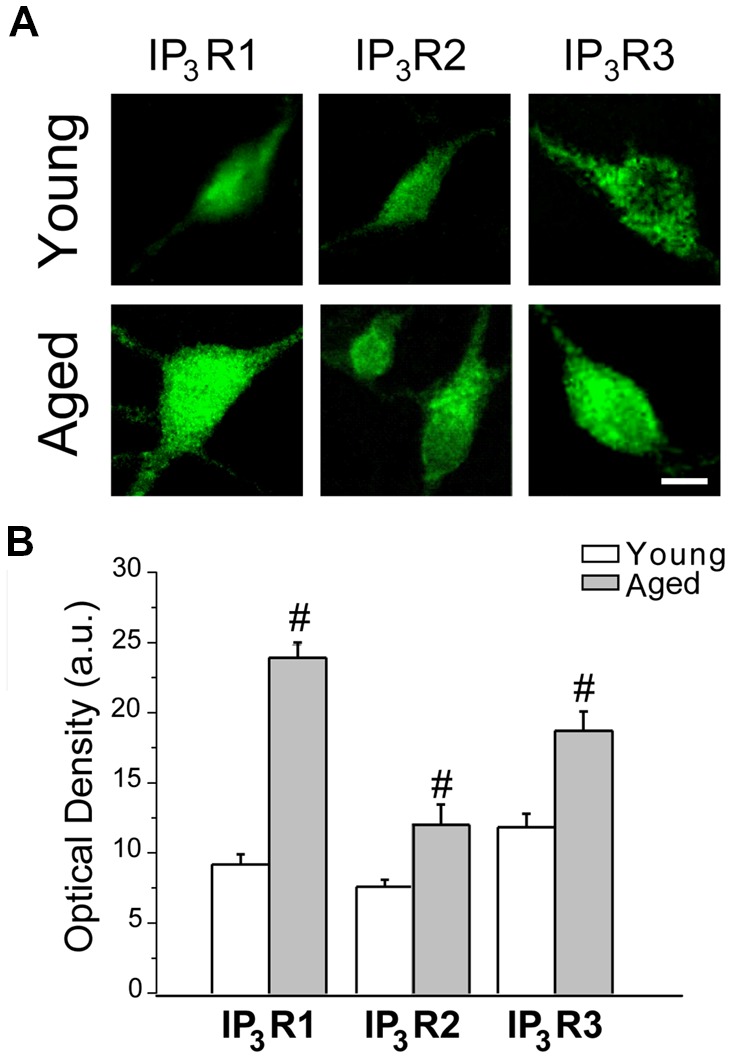
IP_3_ receptor expression increases in aged rat hippocampal neurons. Immunostaining against IP_3_R1, IP_3_R2 and IP_3_R3 was assessed in short-term and long-term cultured hippocampal neurons representing young and aged neurons. **(A)** Representative images of the fluorescence detection of the three isoforms in short-term (young) and long-term (aged) cultured hippocampal neurons. Bar represents 10 μm and applies to all images. **(B)** Quantitative analysis of fluorescence intensity (optical density, arbitrary units) of the three IP_3_R isoforms. Bars represent average ± SEM. *n* = 34, 15, 39, 4, 29 and 14 cells, respectively from three independent cultures. ^#^*p* < 0.05 aged vs. young neurons.

Ca^2+^ release from intracellular stores takes place after activation of IP_3_ receptors at the ER. In addition, IP_3_ receptors are an ER component of the MAMs (Hedskog et al., 2013). Therefore, these receptors are critical to the coupling between ER and mitochondria. Moreover, IP_3_ receptors have been involved in the enhanced cytosolic [Ca^2+^] induced by oligomers in AD (Ferreiro et al., 2004; Demuro and Parker, 2013). Accordingly, we tested whether expression of the three IP_3_ receptor isoforms (IP_3_R1, IP_3_R2 and IP_3_R3) changes during *in vitro* aging. Figure 9A shows representative immunofluorescence images of all three IP_3_ receptor isoforms obtained in short-term (young) and long-term (aged) cultured hippocampal neurons. Figure 9B shows the quantitative analysis of fluorescence intensity (optical density, arbitrary units) corresponding to expression of all three IP_3_R isoforms in young and aged neurons. Results show that optical density of all three IP_3_R isoforms is significantly increased in aged neurons relative to young neurons. These results indicate that changes in expression of IP_3_ receptors may contribute to enhanced response to Aβo in aged neurons and enhanced ER-mitochondria cross talking in aging.

### Aging and Aβ Oligomers Increase ER-Mitochondrial Colocalization in Rat Hippocampal Neurons

**Figure 10:**
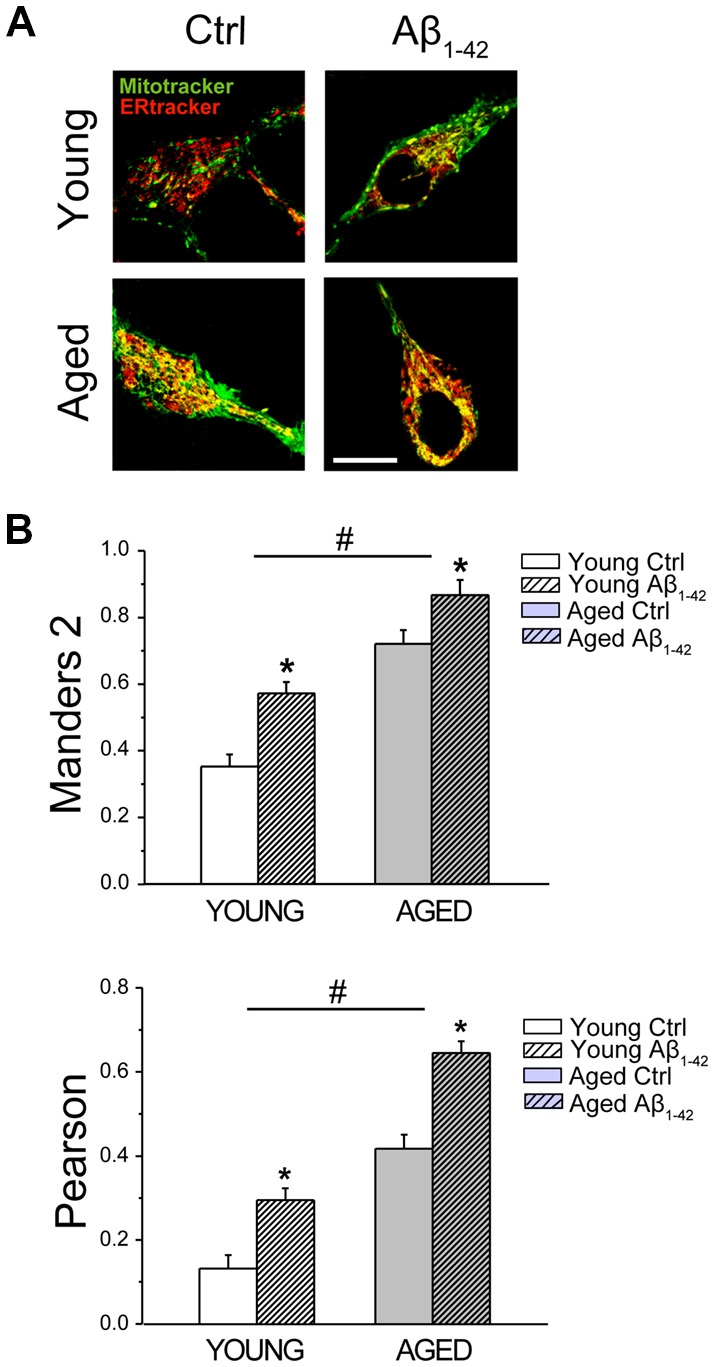
Aging and Aβo increase ER–mitochondria colocalization in rat hippocampal neurons. Short-term (young) and long-term (aged) hippocampal cultures were exposed for 24 h to Aβo (1 μM) or vehicle. Then, cells were incubated with mito-tracker green and ER-tracker red and mitochondrial—ER colocalization was assessed by confocal fluorescence imaging. **(A)** Pictures show representative confocal fluorescence images of short-term (young) and long-term (aged) cultures of rat hippocampal neurons treated with vehicle (Ctrl) or Aβ_1–42_ oligomers. Bar represents 10 μm and applies to all images. **(B)** Bars represent Manders2 and Pearson colocalization coefficients (mean ± SEM) obtained in ROIs corresponding to identified young and aged neurons treated with vehicle (Cntrl) or Aβ_1–42_. Data corresponds to three experiments. *p < 0.05 vs. control group. ^#^*p* < 0.05 vs. young neurons.

We investigated ER-mitochondria colocalization using confocal fluorescence imaging of young and aged rat hippocampal neurons loaded with both MitoTracker green and ER-Tracker red. Figure 10A shows representative confocal images of signals in the green and the red channels of hippocampal neurons treated with vehicle or Aβo 1 μM for 24 h. For colocalization analysis of ER and mitochondria, we computed the Manders2 and Pearson coefficients. Figure 10B shows Manders2 and Pearson coefficients of colocalization of ER and mitochondria. Results show that colocalization coefficients increase significantly in long-term cultured neurons corresponding to aged neurons relative to short-term cultured neurons representing young neurons. In addition, coefficients are further enhanced in neurons treated with Aβo. These results indicate that both aging and Aβo enhance ER-mitochondria colocalization.

The above results may contribute to explain how Aβo increase Ca^2+^ transfer from ER to mitochondria in young neurons (Figure 7). However, they cannot explain why this transfer is rather impaired in aged neurons treated with Aβo. To address this issue, we investigated next the effects of aging and Aβo on mitochondrial membrane potential and ROS generation in rat hippocampal neurons.

### Aging and Aβ Oligomers Decrease Mitochondrial Membrane Potential in Rat Hippocampal Neurons

**Figure 11:**
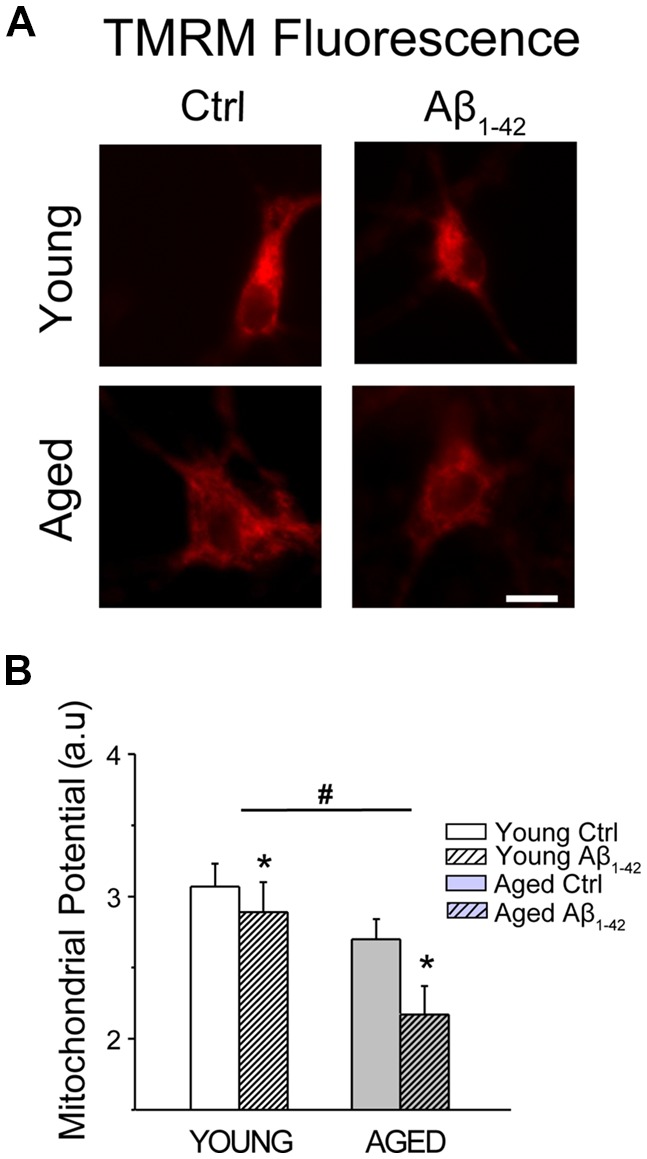
Aging and Aβo decrease mitochondrial membrane potential in rat hippocampal neurons. Short-term (young) and long-term (aged) hippocampal cultures were exposed for 24 h to Aβo (1 μM) or vehicle. Then, cells were incubated with TMRM and mitochondrial membrane potential was assessed by fluorescence imaging. **(A)** Pictures show representative fluorescence images of short-term (young) and long-term (aged) cultures of rat hippocampal treated with vehicle (Ctrl) or Aβ_1–42_ oligomers. Scale bar represents 10 μm and applies to all images. **(B)** Bars represent mean ± SEM values of TMRM fluorescence intensity in selected ROIs corresponding to identified young and aged neurons treated with vehicle (Cntrl) or Aβo. Data correspond to three independent experiments. *p < 0.05 vs. control group. ^#^*p* < 0.05 vs. young neurons.

We have shown previously that *in vitro* aging is associated to a loss of mitochondrial membrane potential in rat hippocampal neurons (Calvo-Rodríguez et al., 2016b). Here, we tested whether Aβo influence also mitochondrial membrane potential. For this end, short-term and long-term cultures of rat hippocampal neurons corresponding to young and aged neurons, respectively, were treated with vehicle or Aβo (1 μM, 24 h). Then, cells were loaded with the mitochondrial potential probe TMRM and subjected to fluorescence imaging. Figure 11A shows representative TMRM fluorescence images of young and aged neurons treated with vehicle or Aβo. Bars in Figure 11B show average (mean ± SEM) optical density values of ROIs corresponding to hippocampal neurons. Results confirm that mitochondrial membrane potential decreases in aged neurons relative to young ones. In addition, the results show that Aβo decrease it further in young and aged neurons. The effect of Aβo on mitochondrial membrane potential is relatively larger in aged neurons than in young ones. The loss of mitochondrial membrane potential favors PTPm, a process that is also enhanced by ROS generation. Accordingly, we investigated next the generation of ROS in young and aged neurons either treated or not with Aβo.

### Aβ Oligomers Increase ROS Generation in Aged Rat Hippocampal Neurons

**Figure 12:**
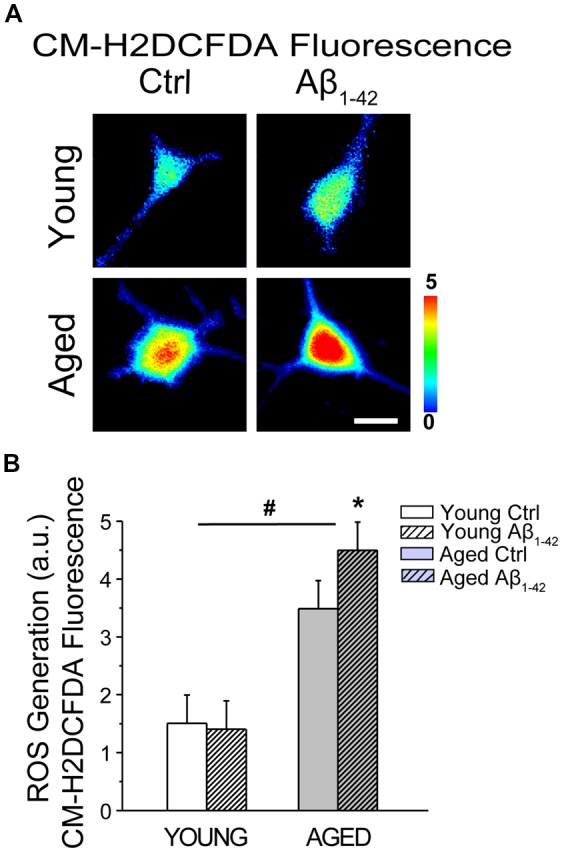
Aging and Aβo enhance reactive oxygen species (ROS) generation in rat hippocampal neurons. Short-term (young) and long-term (aged) hippocampal cultures were exposed for 24 h to Aβo (1 μM) or vehicle. Then, cells were incubated with CM-H2DCFDA and ROS generation was assessed by fluorescence imaging. **(A)** Pictures show representative CM-H2DCFDA fluorescence images coded in pseudocolor of short-term (young) and long-term (aged) cultures of rat hippocampal treated with vehicle (Ctrl) or Aβ_1–42_ oligomers. Scale bar represents 10 μm and applies to all images. **(B)** Bars represent mean ± SEM values of CM-H2DCFDA fluorescence intensity in selected regions of interest (ROIs) corresponding to identified young and aged neurons treated with vehicle (Control) or Aβo. Data corresponds to three experiments. *p < 0.05 vs. control group. ^#^*p* < 0.05 vs. young neurons.

We tested whether aging and Aβo influence also ROS generation in rat hippocampal neurons. For this end, short-term and long-term cultures of rat hippocampal neurons representing young and aged neurons, respectively, were treated with vehicle or Aβo (1 μM, 24 h). Then cells were loaded with the ROS probe CM-H2DCFDA and subjected to fluorescence imaging. Figure 12A shows representative fluorescence images of young and aged neurons treated with vehicle or Aβo. Bars in Figure 12B show average (mean ± SEM) optical density values for ROIs corresponding to hippocampal neurons. The results show that aging increases significantly CM-H2DCFDA fluorescence consistently with enhanced ROS generation in aged neurons relative to young ones. In addition, we found that Aβo increase ROS generation further in aged neurons, having no effect in the young ones. These results are consistent with detrimental effects of Aβo in aged neurons but not in young ones.

**Figure 13:**
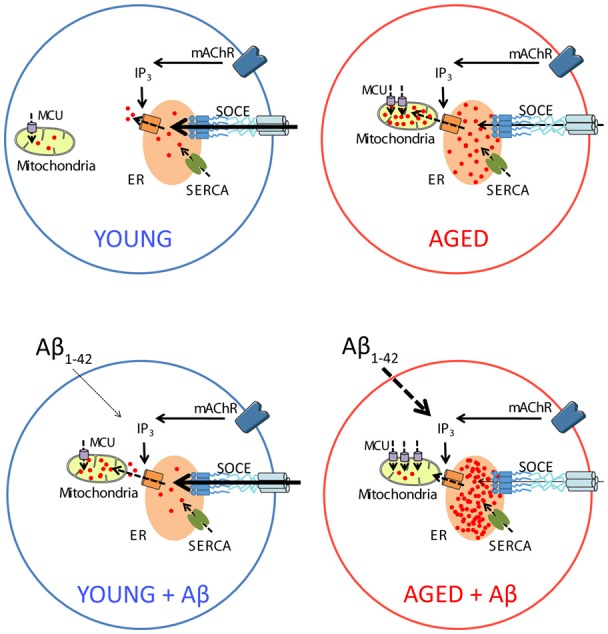
Schematic representing the effects of Aβo on intracellular Ca^2+^ homeostasis in young and aged neurons *in vitro*. In young neurons (top left), activation of metabotropic ACh receptors induce IP_3_ synthesis and release of Ca^2+^ from intracellular stores in the ER. Depletion of Ca^2+^ stores activate SOCE that rise intracellular Ca^2+^ to refill Ca^2+^ stores after activation of the sarcoplasmic and ER Ca^2+^ ATPase (SERCA). Ca^2+^ release is also taken by mitochondria through the MCU for activation of mitochondrial metabolism. Treatment with Aβo increases Ca^2+^ transfer from ER to mitochondria in young neurons without altering any other parameter or promoting apoptosis (bottom left). Aging increases Ca^2+^ store content at the ER, MCU expression and Ca^2+^ transfer from ER to mitochondria but decreases SOCE (top right). Treatment of aged neurons with Aβo exacerbates the increases in Ca^2+^ store content and MCU expression as well as SOCE loss. However, in contrast to young neurons, treatment with Aβo decreases Ca^2+^ transfer from ER to mitochondria in aged neurons and promotes apoptosis (bottom right).

Accordingly, the present results show that Aβo enhance ER-mitochondrial coupling in short-term cultured hippocampal neurons corresponding to young neurons but this is not associated to a detrimental effect. In contrast, in long-term cultured hippocampal neurons, corresponding to aged neurons, Aβo also increases ER-mitochondrial colocalization, but this effect is associated to loss of ER-mitochondrial coupling and detrimental effect (Figure 13).

## Discussion

Signaling by Ca^2+^ plays a fundamental role in learning and memory processes, and participates in survival and neuronal death. Intracellular Ca^2+^ dyshomeostasis has been extensively related to many neurodegenerative diseases, including AD (LaFerla, 2002; Bezprozvanny and Mattson, 2008). Ca^2+^ pathology in AD involves altered Ca^2+^ influx processes as well as Ca^2+^ release from the internal stores and impaired Ca^2+^ buffering capacity. Furthermore, sustained up-regulation of Ca^2+^ levels could both initiate and accelerate the features of AD, including plaque deposition and synaptic loss (Stutzmann, 2007), although if Ca^2+^ dysregulation is a cause or a consequence of AD is still under debate.

Here, we show that chronic exposure to Aβo exacerbates largely the remodeling of subcellular Ca^2+^ associated to *in vitro* aging in cultured hippocampal neurons. First, we show that Aβo increase further basal [Ca^2+^]_cyt_ and spontaneous and synchronous [Ca^2+^]_cyt_ oscillations. These results are consistent with previous reports *in vivo* showing that brains of AD patients exhibit increased [Ca^2+^]_cyt_ levels (Murray et al., 1992). Moreover, recent *in vivo* studies using intravital Ca^2+^ imaging of APP/PS1 transgenic mouse, a mouse model of familial AD, show that basal [Ca^2+^]_cyt_ levels are elevated significantly in approximately 35% of neurites located in areas in close proximity to amyloid plaques (Kuchibhotla et al., 2008). A possible explanation for the increase in the basal [Ca^2+^]_cyt_ levels is the ability of the Aβo to generate ROS, disturbing the function of the membrane ATPases and, thus leading to an increase in the resting [Ca^2+^]_cyt_ level (Mark et al., 1995). Previous studies have shown that the increase in the basal [Ca^2+^]_cyt_ relates to the increase in neuronal excitability and, therefore, with circuit connectivity (Catterall and Few, 2008). Therefore, it could be also speculated that chronic treatment with Aβo increases the excitability of the circuit, as shown by our own *in vitro* experiments, and previously by other groups that use *in vivo* imaging in AD mouse models (Busche et al., 2008, 2012; Šišková et al., 2014). Interestingly, the risk of epileptic activity is particularly high in AD patients with early-onset dementia and during early stages of the disease, which a much higher incidence of crises compared to the reference population of the same age (Amatniek et al., 2006).

SOCE, also called capacitative Ca^2+^ entry, is a Ca^2+^ influx pathway activated after the release of Ca^2+^ from the ER in order to replenish intracellular stores that subserves many different cell and physiological functions in a large variety of cell types (Parekh and Putney, 2005). In neurons, SOCE is involved in synaptic processes as well as neuronal excitability (Moccia et al., 2015). In addition, it has been described to be extensively altered in neurodegenerative disorders (Secondo et al., 2018). The components that participate in SOCE are also dysregulated in neurodegenerative disorders. We have previously shown that SOCE decreases with *in vitro* aging, and this effect is at least partially due to downregulation of Stim1 and Orai1 (Calvo-Rodríguez et al., 2016b). Here, we show that exposure of cultured hippocampal neurons to Aβo has a further detrimental effect on SOCE in the aged neurons without having significant effects in young neurons. Previous work by Tong et al. (2016) using cultured hippocampal neurons expressing mutant PS1 proposed that mutations in PS1 enhance the activity of γ-secretase, thus increasing the cleavage of Stim1 and therefore reducing the activation of Orai1, and SOCE. These effects disrupted dendritic spines arguing that PS1 mutations could contribute to memory loss through SOCE dysregulation. Consistently, treatment of AD mouse models with the SOCE positive modulator NSN21778 stimulates SOCE in the spines and rescues hippocampal long-term potentiation impairment (Zhang et al., 2016). In addition, inasmuch as SOCE contributes to ER Ca^2+^ refilling, it could be reasoned that SOCE downregulation in the aged neurons, and in aged neurons exposed to Aβo, is a compensatory effect to the enhanced filling state of intracellular stores in aging and Aβo treated neurons. In any case, SOCE is strongly downregulated in aged neurons and this process is further exacerbated by Aβo exposure. Interestingly, this effect of Aβo is only statistically significant in aging neurons suggesting that a permissive mechanism associated to aging is involved in the mechanism linking Aβo to SOCE downregulation.

PSs are integral proteins located in the ER membrane that may form low conductivity channels in the ER, known as *leak* channels. These channels may release Ca^2+^ in a passive and constant manner from the ER into the cytoplasm (Tu et al., 2006). Work on transgenic mice has shown that mutations in these proteins, as it occurs in familial AD, result in loss of function of the leak channel activity, causing an increase in the Ca^2+^ level on the intracellular stores (Nelson et al., 2007). We have previously shown that Ca^2+^ store content is significantly increased in aged hippocampal neurons (Calvo-Rodríguez et al., 2016b). Our present data confirm these previous results. In addition, our results show that the chronic treatment of hippocampal neurons *in vitro* with the toxic species of AD, Aβo, increases Ca^2+^ store content further, thus proving that chronic exposure to Aβo causes a similar effect than mutations in PS. These results are supported by previous experiments in PC12 cells exposed to chronic treatment (24 h) with Aβ_1–42_, where an excess release of Ca^2+^ from intracellular stores was shown after stimulation with thapsigargin in free calcium media (Pannaccione et al., 2012). This effect is only observed in long-term cultured hippocampal neurons exposed to Aβo, proving the resistance of the short-term cultured ones to the effects of Aβo. Interestingly, a similar tendency has recently been shown by Lerdkrai et al. (2018). These authors found significant differences in the filling state of the intracellular Ca^2+^ stores in AD compared with WT mice *in vivo*, mainly in hyperactive AD cells. In accordance with this line, the chronic exposure of cultured hippocampal neurons to the Aβo notably increases the peak of [Ca^2+^]_cyt_ induced by both ACh and Caff. This is again pointing to an increased concentration of Ca^2+^ in the ER after exposure to Aβo. Consistently, previous studies in fibroblast from AD patients (Gibson et al., 1996) or in cells carrying the mutated human PS1 (Stutzmann, 2007), showed an abnormal Ca^2+^ release through IP_3_Rs. In addition, aberrant Ca^2+^ increases mediated by IP_3_ in fibroblasts of asymptomatic members of families with AD have also been described (Etcheberrigaray et al., 1998). Other studies have also implicated the RyRs as responsible for this increased release of intracellular Ca^2+^ in animals carrying the PS1 mutation (Chan et al., 2000). Also, Aβ_1–42_ peptides could be responsible for an increase of the isoform RyR3 in transgenic mouse models of AD (Supnet et al., 2006), influencing the amount of Ca^2+^ that is released from the stores when they are activated. Therefore, Ca^2+^ store content is increased in aged neurons and this process is further enhanced by Aβo. Again, Aβo do not influence Ca^2+^ store content in young neurons.

Mitochondria play also an important role in intracellular Ca^2+^ homeostasis by shaping neuronal Ca^2+^ signaling. Mitochondria and ER are structurally and functionally connected in contact points enriched in MAMs (Csordás and Hajnóczky, 2009; Hayashi et al., 2009). MAMs play different roles in cholesterol ester synthesis, phospholipid transport and Ca^2+^ transfer from ER to mitochondria. It has been previously shown that MAM function is altered in the pathology of AD. Area-Gomez et al. (2012) showed a few years ago that there is an enhanced ER-mitochondria connectivity in AD patients of sporadic and familial AD. Consistently, we reported recently that *in vitro* aging is associated to enhanced Ca^2+^ transfer from ER to mitochondria in rat hippocampal neurons (Calvo-Rodríguez et al., 2016b). This view was supported by two findings. First, the increased rise in mitochondrial [Ca^2+^] induced by ACh in Ca^2+^ free medium in aged neurons compared to young neurons. Second, the increased rise in [Ca^2+^]_cyt_ induced by ACh in the presence of the mitochondrial uncoupler FCCP in aged neurons compared to young neurons (Calvo-Rodríguez et al., 2016b). Our present results confirm these previous findings that were carried out using aequorin targeted to mitochondria using an alternative probe for mitochondrial Ca^2+^, Rhod-5N.

Interestingly, we show now that Aβo enhance Ca^2+^ transfer from mitochondria to ER in young neurons. This view is supported by the finding that Aβo enhance the rise in [Ca^2+^]_mit_ elicited by Ca^2+^ release induced by ACh without increasing either the resting levels of [Ca^2+^]_cyt_, the Ca^2+^ store content or the fraction of cells responsive to ACh. This effect is also independent of changes in the expression of MCU, the calcium channel involved in mitochondrial Ca^2+^ uptake. Thus, the most likely explanation for our results is that Aβo oligomers reinforce the relative position and/or coupling between ER and mitochondria. Consistently with this view, it has been shown that Aβ peptide increases inositol-1,4,5-triphosphate receptor and voltage-dependent anion channel protein expression and elevates the number of ER-mitochondria contact points and mitochondrial calcium concentrations in cultured hippocampal neurons (Hedskog et al., 2013). Unfortunately, the time in culture of these neurons was not reported. In these lines, we show here that expression of all three IP_3_ receptor isoforms, including IP_3_R1, IP_3_R2, and IP_3_R3, is increased in aged neurons, which may enhance ER-mitochondrial coupling in aging. In addition, confocal imaging of living neurons and colocalization analysis indicated increased ER-mitochondria colocalization in aged neurons relative to young neurons. Moreover, colocalization was intensified by Aβo in both young and aged neurons. The effects of Aβo on ER—mitochondria coupling in young neurons are not necessarily detrimental since they may favor increased Ca^2+^ transfer between ER and mitochondria to support increased energy demands and Aβo do not induce apoptosis in young neurons, suggesting a physiological role for Aβo in the modulation of ER—mitochondria coupling.

Surprisingly, and in striking contrast with the results in young neurons, exposure of aged neurons to Aβo decreased rather than increased the rise in [Ca^2+^]_mit_ induced by ACh. These results cannot be explained by lack of ACh receptors as [Ca^2+^]_cyt_ responses to ACh are indeed enhanced in aged neurons treated with Aβo. They cannot be explained either by depletion of Ca^2+^ stores since Ca^2+^ stores are actually enhanced in aged neurons treated with Aβo. Finally, lack of Ca^2+^ transfer from ER to mitochondria in aged neurons treated with Aβo cannot be attributed either to MCU downregulation as MCU expression is actually enhanced in aged neurons treated with Aβo. These results suggest that, contrary to our expectations, Aβo nearly abolish ER—mitochondria cross talk in aged neurons and this effect, together with exacerbated remodeling of intracellular Ca^2+^ induced by aging, is highly detrimental as Aβo induces apoptosis to a large fraction of aged neurons.

It is well established that an adequate flux of Ca^2+^ transfer from ER to mitochondria is required to maintain the Krebs cycle (Cárdenas et al., 2010). Thus, loss of Ca^2+^ transfer from ER to mitochondria in aged neurons treated with Aβo may compromise OXPHOS activity leading to mitochondrial damage and collapse of the mitochondrial potential. However, excess of Ca^2+^ transfer associated to enhanced Ca^2+^ store content may lead to mitochondrial Ca^2+^ overload.

Several studies by others have proposed that mitochondrial dysfunction is an early event the pathology of AD. Yao et al. (2009) described mitochondrial bioenergetics deficits before plaque deposition in females in a mouse model of AD. By using Thy-1 APP mice, an AD model that deposits plaques at 6 months of age, Hauptmann et al. (2009) showed mitochondrial dysfunction associated with higher levels of ROS, including mitochondrial membrane potential decrease and reduced ATP levels as soon as 3 months of age, when extracellular deposits are not present. In AD patients, low glucose metabolism at baseline and decline of glucose metabolism is used for monitoring changes in cognition and functionality in AD and mild cognitive impairment (MCI; Landau et al., 2011; Shokouhi et al., 2013). Also, oxidative stress, solidly related to mitochondrial dysfunction, has been proposed before plaque deposition in AD mouse models (Praticò et al., 2001; Rönnbäck et al., 2016) and before clinical symptoms in AD patients (Mosconi et al., 2008). Consistently with previous results (Calvo-Rodríguez et al., 2016b), we show here that *in vitro* aging is associated to a partial loss of mitochondrial membrane potential and this loss is further decreased by Aβo in both young and aged neurons. The effect is mild in young neurons. However, aged neurons treated with Aβo display a quite large loss of mitochondrial membrane potential, thus reducing rather dramatically the driving force for mitochondrial Ca^2+^ uptake. In addition, we also found that aged neurons show increased ROS generation compared with young neurons. Moreover, Aβo increase further ROS generation in aged neurons having no effect in young neurons. Accordingly, loss of mitochondrial membrane potential and enhanced ROS generation induced by Aβo in aged neurons may constrain Ca^2+^ transfer from ER to mitochondria.

In summary, we show that Aβo modulate differentially ER–mitochondria cross talk in young and aged neurons. In young neurons, Aβo increase ER–mitochondria coupling without having a detrimental effect. In contrast, in aged neurons, although Aβo increase also ER-mitochondria colocalization, the loss of mitochondrial membrane potential and enhanced ROS generation impairs Ca^2+^ transfer from ER to mitochondria despite Ca^2+^ stores are overloaded and the expression of molecular players involved in Ca^2+^ transfer increases, thus leading to mitochondrial damage and apoptosis. It has been reported that MAM-associated proteins are up-regulated in AD brain and APP Swe/Lon mouse models (Hedskog et al., 2013), and this effect is mirrored by nanomolar concentrations of Aβ-peptide. Changes in MAM associated proteins in AD could be a rescue response to dysfunctional ER-mitochondria coupling. However, in the face of increased Ca^2+^ store content associated to age and/or AD-related mutations, the further rise in ER-mitochondria coupling likely induced by Aβo may somehow collapse the own coupling leading to neuron damage. Further research is required to address this critical question. In addition, all the above results have been obtained in identified neurons in an *in vitro* model of neuronal aging. As stated above, the fraction of glial cells in hippocampal cultures increases with *in vitro* aging. Whether glial cell may contribute to Ca^2+^ remodeling in aged neurons treated with Aβo remains to be established.

Taken together, our data show that intraorganellar Ca^2+^ remodeling is a hallmark of the aging that is exacerbated in the presence of Aβo. Aging decreases SOCE required for spine stability and increases Ca^2+^ store content and MCU and IP_3_ receptors expression that favors Ca^2+^ transfer from ER to mitochondria. Most of these effects are exacerbated by Aβo but only in aged neurons. In contrast, in young neurons Aβo increase ER-mitochondrial colocalization and Ca^2+^ transfer from ER to mitochondria without having effects on ROS generation or apoptosis and only a minor decrease in mitochondrial membrane potential, probably related to increased use of this potential for ATP synthesis. However, the effect of Aβo may become highly detrimental in the aged neuron and the mechanism for this switch may play a fundamental role in AD and warrants further consideration.

## Author Contributions

MC-R, LN and CV designed the study and wrote the manuscript. MC-R and EH-P performed research and analyzed data.

## Conflict of Interest Statement

The authors declare that the research was conducted in the absence of any commercial or financial relationships that could be construed as a potential conflict of interest.
